# Photo-Crosslinkable Colloids: From Fluid Structure and Dynamics of Spheres to Suspensions of Ellipsoids

**DOI:** 10.3390/gels2040029

**Published:** 2016-11-16

**Authors:** Avner P. Cohen, Maria Alesker, Andrew B. Schofield, David Zitoun, Eli Sloutskin

**Affiliations:** 1 Physics Department and Institute of Nanotechnology & Advanced Materials, Bar-Ilan University, Ramat-Gan 5290002, Israel; avnerco@gmail.com; 2 Department of Chemistry and Institute of Nanotechnology & Advanced Materials, Bar-Ilan University, Ramat-Gan 5290002, Israel; krilovm1@yahoo.com (M.A.); David.Zitoun@biu.ac.il (D.Z.); 3 The School of Physics and Astronomy, University of Edinburgh, Edinburgh EH9 3FD, UK; abs@ph.ed.ac.uk

**Keywords:** photo-crosslinkable colloids, dynamic light scattering, differential dynamics microscopy, ellipsoid, PMMA

## Abstract

Recently-developed photo-crosslinkable PMMA (polymethylmethacrylate) colloidal spheres are a highly promising system for fundamental studies in colloidal physics and may have a wide range of future technological applications. We synthesize these colloids and characterize their size distribution. Their swelling in a density- and index- matching organic solvent system is demonstrated and we employ dynamic light scattering (DLS), as also the recently-developed confocal differential dynamic microscopy (ConDDM), to characterize the structure and the dynamics of a fluid bulk suspension of such colloids at different particle densities, detecting significant particle charging effects. We stretch these photo-crosslinkable spheres into ellipsoids. The fact that the ellipsoids are cross-linked allows them to be fluorescently stained, permitting a dense suspension of ellipsoids, a simple model of fluid matter, to be imaged by direct confocal microscopy.

## 1. Introduction

Colloids, micron-size particles suspended in a solvent, are ubiquitous in nature and technology and may serve as a simple physical model for the phase behavior of atoms and molecules. Colloids minimize their free energy similar to atoms and molecules, yet they undergo a hydrodynamically-overdamped Brownian motion, very different from the ballistic dynamics exhibited by atomic and molecular systems. With the size of an individual colloidal particle being about one micron, there are almost $10^{12}$ of such colloids suspended in 1.0 mL of a dense colloidal fluid. Therefore, these systems constitute a unique source of experimental data, bridging between the behavior of individual particles and the thermodynamics of truly macroscopic systems. Fast modern confocal laser-scanning microscopes allow the structure and dynamics of ~$10^{5}$ individual colloidal particles in a dense fluid to be followed in real-time and in three spatial dimensions. This unique combination of optical microscopy and colloids is very well known and, in fact, these were Perrin’s optical measurements of Brownian diffusion of colloids [1], which provided the first unequivocal proof for the existence of atoms and a reliable estimate of the Boltzmann constant [2]. In addition to being sufficiently large for real-time single-particle tracking by optical microscopy, colloids may also have their interparticle interactions tuned to allow the exploration of a system’s phase space, which is a great advantage compared to the conventional atomic and molecular systems.

The local microscopic structure of a fluid of simple spheres is well-studied. However, the constituents of most real-life fluids are non-spherical, with their rotational and translational degrees of freedom coupled. This coupling does not allow the structure of simple dense fluids of non-spherical particles, such as ellipsoids, to be obtained by classical scattering techniques. Thus, the main method for structural studies of such fluids, of a tremendous fundamental importance, is confocal microscopy of colloidal ellipsoids [3,4,5,6,7]. In addition to their value for the fundamental research, ellipsoidal colloids also open new directions in engineering of photonic and phononic metamaterials [8]. However, while many protocols exist for the synthesis of spherical colloids, synthesizing fluorescent ellipsoidal particles is more challenging, particularly when the composition of these particles must allow for their density- and index- matching in a stable solvent, transparent to visible light. A common approach is to first synthesize colloidal PMMA (polymethylmethacrylate) spheres by dispersion polymerization and then to embed these in a polymer matrix, which is stretched at an elevated temperature elongating the particles [9,10,11]. With the stretching carried out above the glass temperature of PMMA, the particles, cooled down in a stretched state, keep their ellipsoidal shape. The subsequent chemical destruction of the matrix releases these ellipsoidal colloids, which can now be used to form a fluid suspension [3,4,7,9,10,12,13,14,15]. Unfortunately, while the initial PMMA spheres are sterically stabilized by a polyhydroxystearic acid (PHSA) polymer brush layer [16], this layer is significantly damaged during the destruction of the polymer matrix. Thus, the ellipsoids are typically unstable against gelation [17] and have to be charged to remain in a fluid state, which complicates the interparticle interactions and the physical understanding of the phase behavior [3,4,7]. In addition, the high-temperature stretching procedure damages the fluorescent dye inside the particles [14], challenging their confocal imaging. It has been recently suggested [18] that stretching of photo-crosslinkable PMMA (PCPMMA) spheres can be performed and then the fluorescent dye and the PHSA steric layer can be fully restored, post-elongation. In particular, the stretched PCPMMA spheres are photo-crosslinked in their ellipsoidal state; then, high-temperature procedures are employed to load the particles with a fluorescent dye and to covalently link PHSA to their surface. Such procedures are impossible with the common PMMA particles, which would turn spherical if heated to a high temperature. In addition, many other possible technological applications for PCPMMA colloids have been proposed in the literature [18]. However, while the suspensions of common PMMA spheres have been extensively studied in the past [19,20,21,22], the physical properties of PCPMMA spheres have not yet been characterized, so the baseline for the future studies of PCPMMA ellipsoids and other promising applications of PCPMMA colloids is missing.

In our current work, we synthesize spherical PCPMMA colloids and, employing several different experimental techniques, fully characterize their size, their size distribution, and also the structure and dynamics of their fluids. For particle synthesis, we employ a protocol which is similar, yet not identical, with the one used in the previous work [18] (see the Experimental section). We suspend the particles in a density- and index- matching solvent, forming a stable suspension, the bulk structure of which is accessible by confocal microscopy. We demonstrate that the particles significantly swell in this solvent. The structure of these suspensions and their dynamics, which we measure by the recently-developed confocal differential dynamic microscopy (ConDDM) [19], indicate that the particles are charged much more strongly than the common sterically-stabilized PMMA colloids in a similar solvent [20,21,23]. Note that while ConDDM has been recently employed for characterization of common PMMA spheres [19], the diffusion coefficients of crowded suspensions were not extracted; the corresponding information for PCPMMA has also been completely missing. Finally, we demonstrate that the particles can be stretched into an ellipsoidal shape, fluorescently-labeled, and resuspended in a solvent for confocal studies.

## 2. Results and Discussion

All details of PCPMMA particle synthesis, photo-crosslinking, fluorescent staining, and preparation of the suspensions are described in the Experimental section. In the following, we describe the characterization of the individual particles and of their suspensions.

### 2.1. Particle Characterization

#### 2.1.1. Electron Microscopy

**Figure 1:**
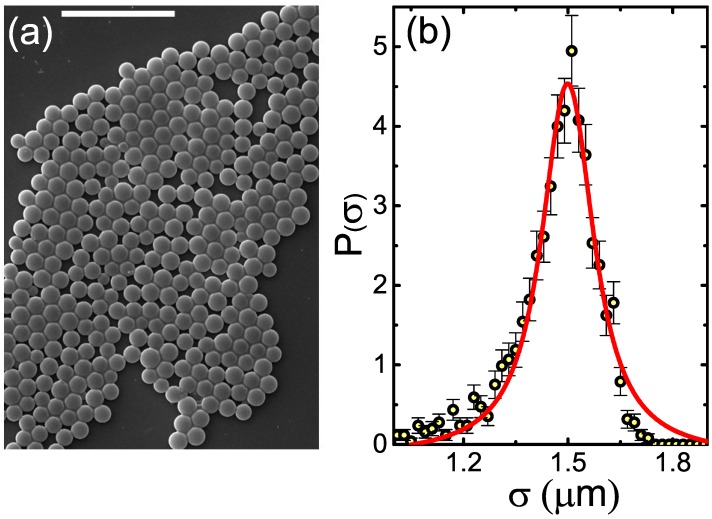
(**a**) SEM image of the original colloidal spheres demonstrates that their polydispersity is relatively low. The scale bar is 10μm; (**b**) The distribution of particle diameters P(σ) (symbols), as obtained by SEM measurements. The **red** curve is a Voigt function fit, shown as a guide to the eye.

In order to characterize the shape of the spherical colloidal particles, we deposit them from hexane onto a clean glass microscopy slide, dry the sample under vacuum, and obtain scanning electron microscopy (SEM) images at 5–30 keV, employing the Quanta Inspect (FEI, Hillsboro, OR, USA) (FEI${}^{\mathrm{TM}}$) setup. A typical image of our initial spherical particles is shown in Figure 1a; note the relatively low polydispersity of the particles. To obtain a quantitative estimate of the particle size distribution P(σ), we obtain the diameters *σ* of >1200 spheres, employing a Circle Hough Transform-based [24] algorithm for automatic detection of all particle radii in SEM images. The resulting P(σ) is closely described by a Voigt function, peaking at σ=1.499±0.002μm (Figure 1b). The apparent polydispersity [25] $\delta \equiv \sqrt{\langle \sigma^{2}\rangle }/\langle \sigma \rangle$ is 0.08. Importantly, *δ* is influenced by the accuracy of *σ* measurements and by the SEM imaging artifacts. Thus, for the relatively small particles studied in the present work, this *δ* value may probably slightly overestimate the true polydispersity of the colloids [26].

#### 2.1.2. Dynamic Light Scattering

**Figure 2:**
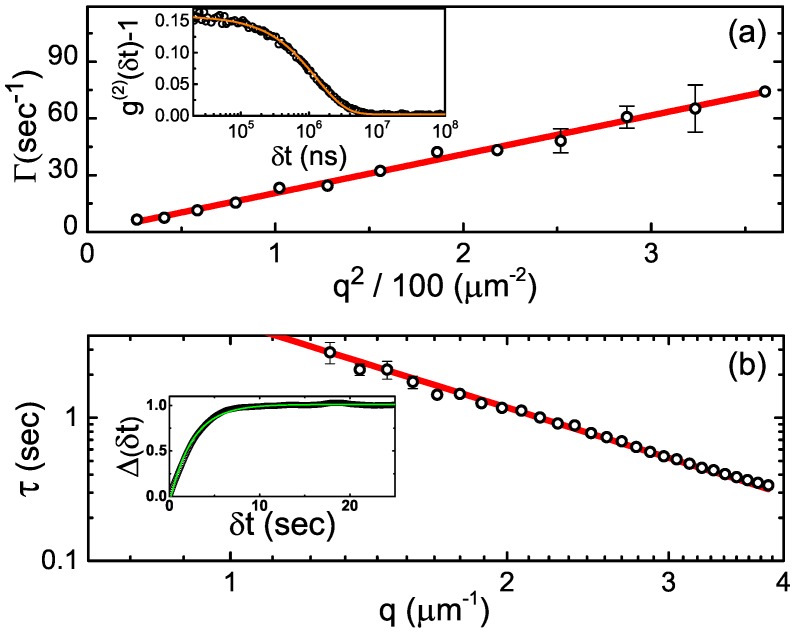
(**a**) Particle sizing by DLS (dynamic light scattering). The experimental decay rate Γ (scatter) of the DLS intensity autocorrelation function $g^{\left(2\right)}\left(\delta t\right)$ is shown to scale linearly with $q^{2}$, indicative of a low particle polydispersity. The **red** line is the theoretical fit, from which the particle diffusion constant is extracted. A representative autocorrelation function (obtained at $\theta =55^{\circ }$) is shown in the inset (scatter); note the perfect match by the theoretical fit (Equation (1), solid curve). (**b**) Particle sizing by ConDDM (confocal differential dynamic microscopy). The correlations between the subsequent images decay over time τ(q), which for the dilute samples is linear in $q^{2}$; note the double-logarithmic scale. The experimental data (scatter) are fitted by the theory (**red** line), allowing the diffusion coefficient to be extracted. The extracted value is in a perfect agreement with the DLS, indicating a significant swelling of the particles in the solvent. The inset shows a typical variation of Δ(δt) (in arbitrary units), which stems from the decay of the correlations between the images. Note the perfect agreement between the experiment (scatter) and the theoretical fit (solid **green** curve).

To characterize the size of the colloids in the suspended state, we use dynamic light scattering (DLS). While our SEM measurements provide the full P(σ), they are carried out in a vacuum, where the particles are possibly shrunk by drying. For DLS measurements, we suspend our colloids in the mixture of decalin and tetrachloroethylene (TCE), as described in the Experimental section. The refractive index of this mixture, at an ambient temperature, was measured as n=1.488, employing the Abbe-2WAJ refractometer (PCE Americas Inc., Palm Beach, FL, USA). The ambient-temperature viscosity was obtained as 1.2 mPa·s, employing a Cannon–Manning semimicro viscometer (CANNON Instrument Company, State College, PA, USA). The DLS measurements were carried out with a Photocor${}^{\mathrm{TM}}$ goniometer-based setup (Photocor Instruments, Tallinn, Estonia), with the time-averaged scattered intensity autocorrelation, $g^{\left(2\right)}\left(\delta t\right)=\langle I\left(t\right)I\left(t+\delta t\right)\rangle$, measured over a wide range of scattering angles *θ*. Such multiangle DLS measurements [19,27] are much more reliable than measurements done with the more common fixed-angle DLS setups, which are only capable of carrying the measurements at a few different *θ*. For perfectly monodisperse particles at a very low particle concentration ($\phi \ll 10^{-3}$),

(1) $$g^{\left(2\right)}\left(\delta t\right)=B+\beta \exp\left(-2\Gamma \delta t\right),$$

where *B*, *β*, and Γ are the baseline, the contrast, and the decay rate [27]; λ=633nm is the radiation wavelength. The particle size information is encoded in Γ, which is a function of the wavevector transfer q=(4πn/λ)sin(θ/2). A typical experimental $g^{\left(2\right)}\left(\delta t\right)$ of our particles is shown in the inset to Figure 2a. Note the perfect fit by the theoretical expression (Equation (1)), confirming that the particle polydispersity is low [27].

To extract the average particle size, we plot Γ as a function of $q^{2}$; a perfectly linear scaling is observed, with no offset at q=0, as demonstrated in Figure 2a. While a denser sampling along the *q*-axis is needed for a quantitative DLS measurement of the polydispersity, the fact that $\Gamma (q^{2})$ is linear indicates that the polydispersity is relatively low [28]. The diffusion coefficient $D_{0}$ of the colloids is the slope [19,27] of $\Gamma (q^{2})$, so that $D_{0}=\Gamma /q^{2}=0.22\pm 0.01$ $\mu m^{2}/s$. The particle diameter is then obtained as $\sigma =k_{B}T/3\pi \eta D_{0}=1.66\pm 0.08$ μm. This value is larger by >10% compared to the SEM-derived particle diameter. The observed discrepancy between SEM and DLS is far larger than the uncertainty of either of these techniques, indicating that the colloids swell in this solvent, significantly increasing in their size compared to the dry state probed by SEM. We note that the swelling of soft materials has recently been used for an exciting superresolution imaging of biological samples, providing an important motivation for characterization of the swelling properties of polymers [29]. To make sure that the particles have swollen to their equilibrium size, we repeated the DLS measurements of the same particles for three days; then, the measurements were also repeated after two weeks. No diameter change was detected in these measurements, indicating that the particles have already equilibrated inside the solvent. Interestingly, particle swelling is independent of the crosslinking; the same *σ* was obtained for both the crosslinked and the non-crosslinked particles.

#### 2.1.3. Confocal Differential Dynamic Microscopy

As an additional test of particle swelling, we employ the confocal differential dynamic microscopy (ConDDM), a recently developed technique, where particle dynamics within the suspension are obtained by real-space microscopy [19,30,31]. With the Rayleigh scattering intensity being proportional to $\sigma^{6}$, the DLS-derived *σ* may be biased, at a finite polydispersity, by the larger particles. The ConDDM measurements, where the signal comes from particle fluorescence, rather than from the Rayleigh scattering, are not subject to such a bias; thus, ConDDM measurements provide, at these very low *ϕ*, an additional test for the *σ* value.

In ConDDM, time series of two-dimensional confocal slices through the suspension are obtained. Pairs of images, separated by a time interval δt, are selected. The images are then subtracted one from the other, removing any time-independent background [19,30,31]. Next, we calculate the 2D Fourier transform of this image difference, square its magnitude, and average the result over all image pairs having the same δt. The radial average of the resulting power spectra Δ(q,δt), the ConDDM variant of $g^{\left(2\right)}$, is proportional to [1−f(δt,q)]+B, where f(δt,q) is equivalent to the intermediate scattering function and *B* is a (very small) background. The experimental Δ(q,δt) are perfectly matched by a theoretical fit (see inset to Figure 2b), where f(δt,q)≡exp[−δt/τ(q)], allowing the characteristic diffusion time τ(q) to be extracted. As for the DLS, $\tau \left(q\right)=\Gamma^{-1}=1/D_{0}q^{2}$; indeed, the correct power law is observed in Figure 2b, where a double-logarithmic scale is used. The resulting $D_{0}=0.21\pm 0.01\;\mu m^{2}/s$ yields σ=1.74±0.08 μm, coinciding, within the statistical error, with the value obtained by DLS. This perfect agreement between the ConDDM- and the DLS- derived particle diameters proves the validity of these methods and also indicates that the size distribution of our colloids is narrow. With the DLS data being strongly biased by the larger particles, as mentioned above, broader size distributions would not allow the same *σ* to be detected by both of these methods.

### 2.2. Dense Fluids: Structure

**Figure 3:**
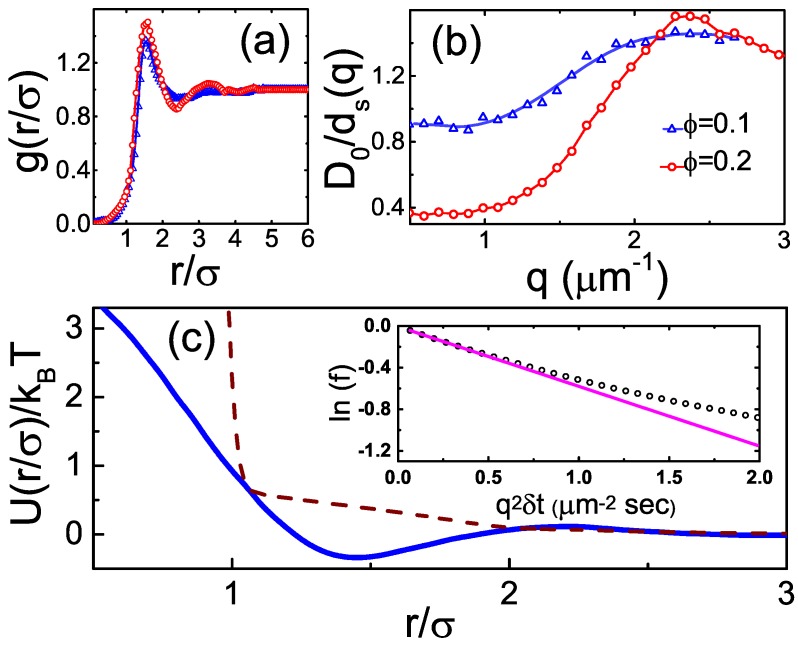
(**a**) The radial distribution function, g(r), at two different volume fractions: ϕ=0.1 (**blue** triangles) and ϕ=0.2 (**red** circles). The *r* values are normalized by *σ*, as obtained by DLS. The lines are guide to the eye. Note that the position of the first peak, occurring much higher than r/σ=1, indicates that the colloids are charged. Similarly, the wide first peak is typical for the charged systems. (**b**) The short-time diffusion rates $d_{s}\left(q\right)$, as obtained by ConDDM. Note, the *y*-axis corresponds to the (dimensionless) reciprocal of the diffusion rate, $D_{0}/d_{s}\left(q\right)$; the lines are guide to the eye. The $d_{s}\left(q\right)$ values are obtained by fitting an exponential to the corresponding f(δt,q), with the fit limited to $\delta t\ll \tau_{R}$, as explained in the main text. A typical fit (**pink** line) is shown in the inset to panel (**c**), where the experimental f(δt,q) appear in **black** symbols. (**c**) The pair potential of our PCPMMA (photo-crosslinkable PMMA) spheres (solid **blue** curve), as obtained by a numerical inversion [34] of the experimental g(r), exhibits a small dip at low *r*, suggesting that slight interparticle attractions may be present. The curve was obtained by averaging over several different *ϕ*, to minimize the numerical noise; the curves at all *ϕ* are very close together, validating this averaging. To test the inversion procedure, we carry out a similar inversion for a theoretical g(r) of hard spheres (**brown** dashes). No attractions are detected in this test case, in a further support of the currently-used numerical protocol [34].

To probe the interparticle potentials of the PCPMMA spheres, we measure the structure of their fluid suspensions. While, by definition, an ideal gas exhibits no particle correlations and the crystals are fully correlated, dense fluids are an intermediate between these two limits, exhibiting short range correlations. The correlations in fluids are a sensitive measure of the interparticle potentials U(r) at a finite *ϕ*. While multiple (relatively-) direct methods exist [32], allowing the colloidal pair potentials at ϕ→0 to be measured, the *ϕ*-indepedence of the pair potential cannot, in general, be guaranteed for the colloids. To characterize the interparticle correlations at a finite *ϕ*, we obtain the radial distribution function g(r) (Figure 3a), using particle center positions detected by microscopy [33]. This function is proportional to the probability for two particles to have their centers separated by a distance *r*. By normalization, the g(r) is 1 for an ideal gas, where the correlations are missing. At small separations r<σ, g(r)→0, due to the mutual exclusion of the colloids. The peaks of g(r) correspond to the liquid coordination shells. The contrast of these shells exhibits an exponential decay, characteristic of the short range order in fluids. Notably, the principal peak of the experimental g(r) occurs much higher than at r=σ, indicating that the particles are electrically charged; thus, their effective particle diameter is higher than either the DLS- or the ConDDM- derived hydrodynamic radii. Indeed, the wide and smooth shape of the principal peak is also typical of the soft charge repulsions.

For a more quantitative estimate of U(r), we invert the experimental g(r) employing the classical Ornstein–Zernike formalism and the hypernetted chain (HNC) approximation. An iterative technique has been proposed [34], allowing for the convergence of U(r) at a finite *ϕ*. The resulting U(r), obtained for the experimental samples in a wide range of *ϕ*, almost fully overlap. In all cases, a potential well is clearly visible at short particle separations (solid curve in Figure 3c). The shape of the U(r) is virtually unchanged when the full three-dimensional g(r) is reconstructed by the algorithm of Wilkinson and Edwards [35] and used for the inversion procedure; accounting for the finite particle polydispersity [36], avoided at present to minimize the generation of numerical noise, may additionally increase the depth of the potential well by ~20%. No similar potential well occurs for the U(r) obtained by an inversion of the theoretical g(r) of the ideal hard spheres (dashes in Figure 3c). These observations suggest that the pair potentials of our colloids include a significant attractive contribution. Very recently, similar attractions have been detected in two-dimensional suspensions of common PMMA colloids and attributed to the presence of dipolar interactions [22]. Additional studies are needed to confirm the physical mechanism of the attractions observed in our current work.

### 2.3. Dense Fluids: Dynamics

To further characterize the properties of the PCPMMA colloids, we measure the dynamics in dense fluids of these particles [21,37]. In general, there are three distinct regimes of dynamics in fluid colloidal suspensions. At the very short times, $t<\tau_{B}$, the dynamics are ballistic; here, $\tau_{B}=m/3\pi \eta \sigma$ is the Brownian relaxation time and *m* is the mass of an individual colloidal particle. At longer times, $\tau_{B}\ll t\ll \tau_{R}$, the dynamics is diffusive; here, $\tau_{R}=\sigma^{2}/4D_{0}$ is the time for a particle to diffuse its own size in a free solvent [38], so that, for $t\ll \tau_{R}$, the direct steric interactions between the colloids are negligible. In this so-called ‘short-time dynamics’ regime ($\tau_{B}\ll t\ll \tau_{R}$), only the solvent-mediated hydrodynamic interactions between the colloids matter. At even longer times, $t\gg \tau_{R}$, the long-time diffusion sets in. In this regime, the dynamics is governed by both the hydrodynamic interactions and the random encounters between the colloids [21]. In our case, we estimate: $\tau_{R}\approx 3$ s and $\tau_{B}\approx 150$ ns. Thus, our ConDDM measurements, carried out at 30 fps, allow the short-time dynamics (δt≤4 frames) to be probed, for a wide range of *q* values.

To obtain only the short-time dynamics contributions, we fit the experimental f(δt,q) by a decaying exponent, limiting the fit to δt≤0.26 s (inset to Figure 3c). Clearly, significant deviations from a simple exponential behavior occur at larger δt, due to the crossover to the long-time diffusion regime. The fitted characteristic time of the f(δt,q)-decay, τ(q), yields the short-time diffusion rate $d_{s}\left(q\right)=1/\left[q^{2}\tau \left(q\right)\right]$. This $d_{s}\left(q\right)$ is a sensitive function of both *q* and *ϕ*, as demonstrated in Figure 3b, where the data are normalized by the free particle diffusion rate $D_{0}$, for non-dimensionalization. As expected [21,37], the $D_{0}/d_{s}\left(q\right)$ is peaking at $q=Q_{m}$, corresponding to the principal peak position of the structure factor S(q). In the real space, this *q*-value represents the most probable interparticle separation, given by the principal peak position $R_{m}$ of the g(r). Indeed, $2\pi /Q_{m}\approx 1.6\sigma$, in full agreement with Figure 3a. Thus, the diffusion is slowed by the liquid coordination shell structure: particles separated by $R_{m}$ are trapped for a longer time in this thermodynamically-favorable configuration.

**Figure 4:**
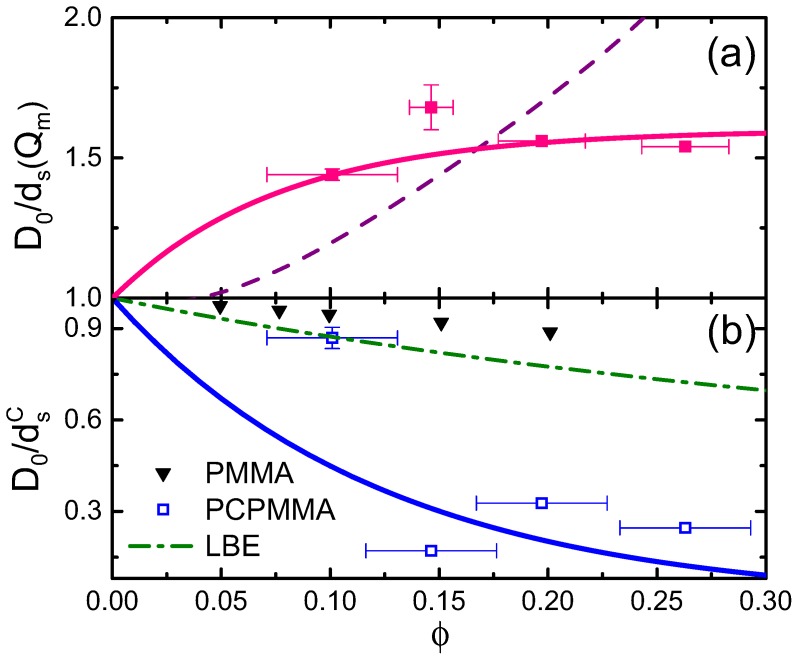
(**a**) The (reciprocal of the) short-time diffusion at $q=Q_{m}$ demonstrates the lifetime of structural fluctuations, with a wavelength corresponding to the interparticle separation. The data, obtained for our PCPMMA fluids by ConDDM (**pink** squares), are shown for a range of different *ϕ*. A polynomial fit to the hard PMMA spheres’ data [21] obtained by DLS (see main text), is shown by a dashed curve. (**b**) The (reciprocal of the) short-time collective diffusion (the limiting behavior at q→0) exhibits a very steep *ϕ*-dependence for our charged PCPMMA spheres (open **blue** squares, obtained by ConDDM). A much less steep behavior was previously obtained for the hard PMMA spheres [21], employing DLS (**black** triangles). The theoretical prediction for the hard spheres (dash-dotted curve, marked as LBE [fluctuating lattice Boltzmann equation method]) [21,43,44] does not match either of the experimental dependencies. The solid lines are guides to the eye. Note, in contrast with the common intuition, both the experimental and the theoretical rates of collective diffusion *speed up* with *ϕ*.

Furthermore, the short-time diffusion rate at $q=Q_{m}$ depends on the colloidal concentration, as demonstrated by squares in Figure 4a. For higher *ϕ*, the structural fluctuations away from the shell structure are energetically more costly, so that the two-particle states, where $r=R_{m}$, are long-lived. Thus, $D_{0}/d_{s}\left(Q_{m}\right)$ is an increasing function of *ϕ*. Remarkably, a much steeper increase with *ϕ* has been observed for the PMMA hard spheres, where the DLS-derived data [21] have been fitted by a polynomial: $D_{0}/d_{s}\left(Q_{m}\right)\approx 1-2\phi +58\phi^{2}-220\phi^{3}+347\phi^{4}$ (dashes in Figure 4a). Moreover, $D_{0}/d_{s}\left(Q_{m}\right)$ values obtained in previous experimental [39,40] and theoretical [41] studies of charged colloids in aqueous suspensions exceed both our current data and the data obtained for the hard spheres. Further theoretical studies are necessary to fully understand the effect of the complex U(r) shape in PCPMMA on dense suspension dynamics; notably, particle porosity has recently been demonstrated to reduce the $D_{0}/d_{s}\left(Q_{m}\right)$ beyond the hard spheres’ limit [42].

Finally, the q→0 limit of $d_{s}\left(q\right)$ represents the collective (short-time) diffusion. Counterintuitively, the corresponding diffusion rates $d_{s}^{C}\left(\phi \right)$, obtained by an extrapolation of the experimental data to q=0, *speed up* with *ϕ*; see Figure 4b. The same trend is also clearly observed by comparing the two data sets, for ϕ=0.1 and ϕ=0.2, in Figure 3b. A similar behavior was observed previously for the hard spheres and attributed to the collective motion of neighboring particles, allowing for a fast decay of the long-wavelength fluctuations [21]. However, as for the $d_{s}\left(Q_{m}\right)$, the *ϕ*-dependence is much steeper in our samples (open squares), compared to both the experimental (triangles) and the theoretical [21,43,44] (dash-dotted curve) hard spheres. While similar trends have been previously detected for purely-repulsive charged colloids in aqueous media [39,40] and also for the porous hard spheres [42], additional experimental and theoretical work is clearly needed to develop a full understanding of these experimental data.

### 2.4. PCPMMA Ellipsoids

**Figure 5:**
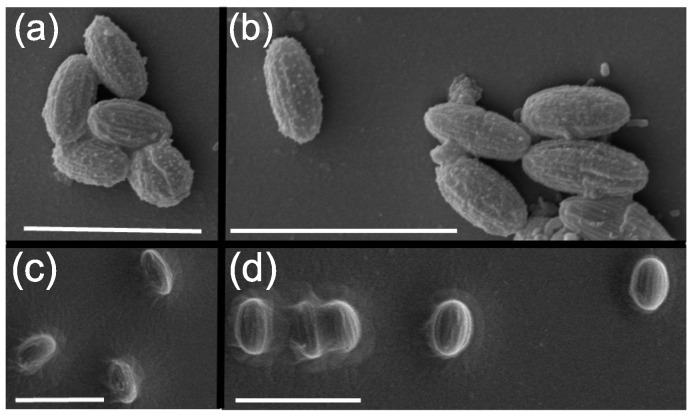
SEM images of prolate ellipsoids, obtained by stretching of the spherical PCPMMA colloids by (**a**,**b**) 90%; and (**c**,**d**) 70%. The scale bar lengths are 5μm.

A great advantage of PCPMMA, compared to the classical PMMA spheres, is that these particles can be fluorescently stained anew, after their stretching into an ellipsoidal shape. With the original dye being (almost completely) bleached by the elevated-temperature stretching process [14], the ability to load new dye into the stretched PCPMMA by the swelling/deswelling procedure is very important. We stretch our PCPMMA colloids into ellipsoidal shapes, as described in the Experimental Section. Particles in one batch are stretched by 70%, while those in the other batch are stretched by 90%. The SEM images (Figure 5) demonstrate a successful formation of ellipsoids in both cases.

**Figure 6:**
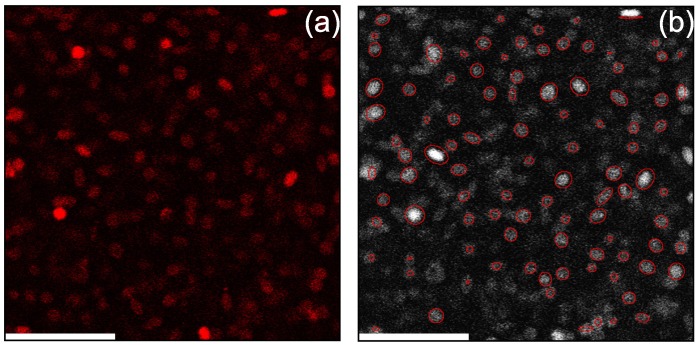
(**a**) A raw confocal slice through a fluid bulk suspension of PCPMMA ellipsoids. Note the brightness of the particles, achieved by a swelling/deswelling fluorescent staining, carried out after particle stretching; such staining is impossible with the conventional PMMA ellipsoids [3,4,7]. While our confocal images deal with a fluid of mobile particles, so that resonant scanning and piezo-z positioning had to be employed, much higher quality images of similar particles have been recently obtained for *static* PCPMMA ellipsoids, residing at the bottom of a sample chamber [18], where much slower galvanometric confocal scanning is possible. (**b**) The same image, with the positions and the orientations of the particles, as detected by our algorithm, marked by **red** ellipses. Note that all particles that are not perfectly parallel to the optical slice appear more rounded than they actually are. The scale bar length is 14μm, in both panels.

With the PCPMMA ellipsoids stained by the swelling/deswelling procedure, we suspend the particles in a mixture of decalin and TCE, as was done for the spheres (see above). The particles, as stretched, are unstable, with the sterically-stabilizing PHSA monolayer partly destroyed by the sodium methoxide (SM). While recoating by PHSA and covalently linking it to particle surface must be possible with the PCPMMA ellipsoids, in the current work, we charge-stabilize the particles instead. For charge stabilization, we introduce AOT (dioctyl sodium sulfosuccinate, Sigma-Aldrich 98%, St. Louis, MO, United States) micelles into the suspension. At AOT concentrations of 70–80 mM, the AOT micelles charge the particles [45], but also screen the long-range Coulomb repulsions, so that the resulting interactions are almost hard [3,4,7]. A typical confocal microscopy slice through the bulk of the suspension is shown in Figure 6a. While a confocal image of PCPMMA ellipsoids deposited from pure decalin onto a glass substrate has been recently demonstrated by Klein et al. [18], confocal images of bulk fluid suspensions of such particles have hitherto not been published. Note the excellent brightness of all the particles, and, importantly, particles which are not parallel to the optical slice appear more rounded than they actually are. Interestingly, some particles appear much brighter than the others. While these variations of brightness do not matter for the particle tracking, the origin of this phenomenon is unclear. We suggest that the brighter particles can potentially be used as tracers in rapid dynamics experiments, where the full trajectories of all particles cannot be tracked.

To locate the positions of all particles, the slice is processed, so that the fluorescent colloids appear as bright, well-separated features on a dark background. A two-dimensional slice through an ellipsoidal particle is an ellipse. The center positions and the angles of orientation of all such ellipses, in each of the two-dimensional slices, are measured employing the covariance matrix formalism [4,7]. The positions and the orientations of the particles, as detected, are marked by red ellipses in Figure 6b; note the very good agreement with the raw data. Finally, the tracked particle positions can be used to obtain the structure of a fluid of ellipsoids. Some preliminary data of this kind are demonstrated in the Appendix, accompanied by a tentative theoretical analysis. Further studies in this direction are underway.

## 3. Experimental

### 3.1. Materials

Methyl methacrylate (MMA), methacrylic acid (MA), octyl mercaptane (OctSH), azobisisobutyronitrile (AIBN), butyl acetate (BA), dodecane, hexane, hydroxy terminated polydimethylsiloxane (PDMS), trimethylsilyl terminated poly(dimethylsiloxane-co-methylhydrosiloxane), tin(II) 2-ethylhexanoate, tetrachloroethylene (TCE, >99.5%), cis/trans decahydronaphthalene (decalin), and rhodamine B chloride (95%) were purchased from Sigma-Aldrich. Ethyl acetate (EA) was obtained from Tedia Company Inc. (Fairfield, OH, USA). Isopropyl alcohol (AR), acetone, and cyclohexanone (>99%) were obtained from Frutarom (Haifa District, Israel), Macron Fine Chemicals (Center Valley, PA, USA), and Sigma-Aldrich, respectively. Sodium methoxide (SM) was obtained from Fluka (>97%) (Sigma-Aldrich). The photo-crosslinking comonomer 2-cinnamoyl oxyethylacrylate (CEA) was supplied by Polysciences (Warrington, PA, USA). The individual poly-12-hydroxystearic acid chains were produced by Azko Nobel (Slough, UK) and were converted into a PMMA-PHSA comb stabilizer at Edinburgh University using a procedure which is described elsewhere [16].

### 3.2. Particle Synthesis

**Figure 7:**
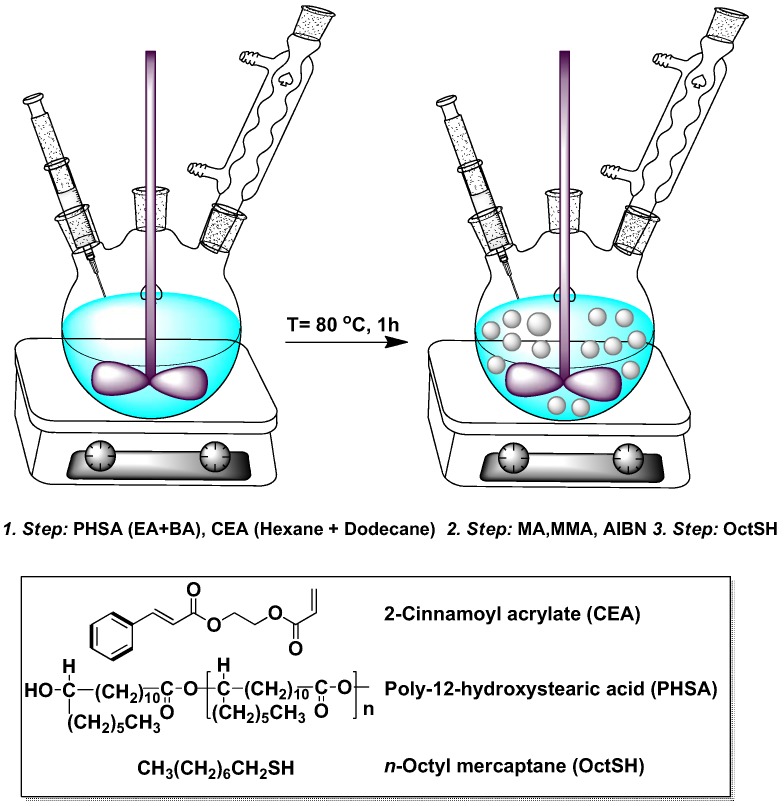
Dispersion polymerization procedure. The materials are introduced into a three-neck round bottom flask in three separate steps. Then, the contents are stirred for 1 h at $80{\;}^{\circ }$C, after which the solid contents of the dispersion are transferred into hexane.

The dispersion polymerization procedure, commonly used for preparation of colloidal PMMA spheres [16], has recently been extended to allow the photo-activated cross-linker 2-cinnamoyl oxyethylacrylate (CEA), to be incorporated into the particles [18]. This cross-linker has the advantage that it can be initiated after the particles have been formed and can be activated at any point in the post-preparation processing. Prior to particle preparation, the MMA monomer is purified by vacuum filtration through alumina. Then, the steric stabilizer solution is prepared by adding the stabilizer [16] (PHSA, 0.31 g, as a powder) into a mixture of BA (0.17 mL) and EA (0.35 mL); vigorous mixing is necessary to have the PHSA fully dissolved. In addition, we also prepare a solvent solution, by mixing hexane (15 mL) with dodecane (6.5 mL), and dissolving MA (0.26 mL), MMA (13.8 mL), CEA (0.763 mL), AIBN (3.93 mL), and OctSH (102 μL) in this solution. Finally, this solution and the steric stabilizer solution are poured into a three-neck round bottom flask (250 mL); see Figure 7, where the individual steps of adding the material into the flask are detailed. The flask, equipped by a reflux condenser, was immersed in an oil-bath, pre-stabilized at $80{\;}^{\circ }$C on top of a digital hot plate. At this temperature, the reaction was allowed to run for 1 h, with the contents of the flask being homogenized by an overhead stirrer. While carrying out the reaction under an inert atmosphere was possible with our current setup, the atmosphere being either inert or not appearing to be unimportant for its success. When the reaction was finished, the solid contents of the dispersion were transferred into hexane by repetitive centrifugation, decantation, and redispersion.

### 3.3. Photo-Crosslinking of the Particles

**Figure A2:**
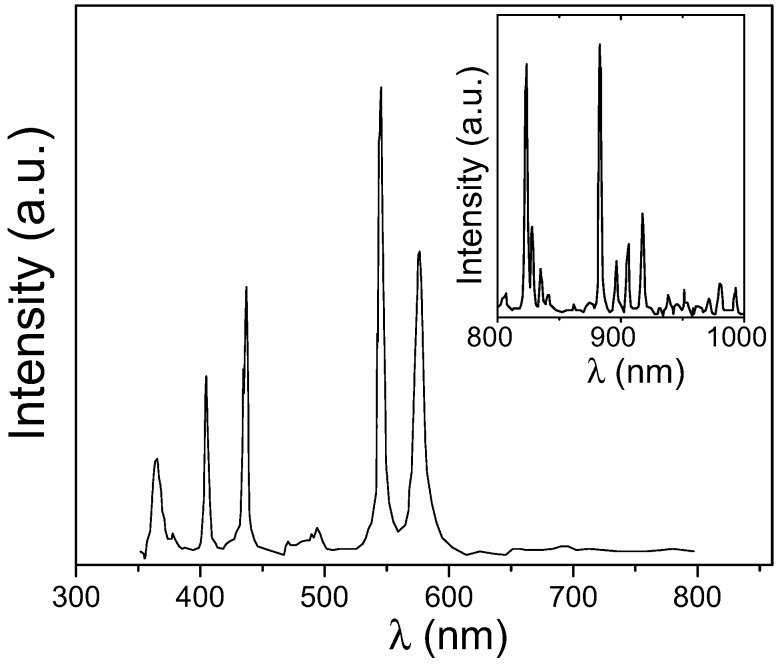
The experimentally-determined spectrum of the mercury–xenon UV lamp employed for the polymerization procedures. **Main panel:** short wavelength part of the spectrum, dominated by the Hg peaks. **Inset:** long wavelength part of the spectrum, dominated by the Xe peaks.

To photoactivate the crosslinker, 400 mg of the particles were dispersed in 20 mL of pure decalin and subjected to UV irradiation. The dispersions were agitated by a magnetic stirring bar, to prevent particle settling. Irradiation was performed by focusing the light of a high pressure mercury–xenon short arc lamp to the middle part of a cuvette for 3 h. The experimentally-determined spectrum of this lamp is shown in Figure A2. To avoid excessive heating of the sample, the cuvette was wrapped around by plastic tubes, through which water at $6{\;}^{\circ }$C was circulated. The UV light passed through the opening of the cuvette, so that the blocking of it by either the plastic tubes or the glass walls of the cuvette was completely avoided.

### 3.4. Stretching of Colloidal PCPMMA Spheres to Form Colloidal Ellipsoids

To form colloidal ellipsoids, we stretch the PCPMMA spheres prior to their photoactivation [3,4,9,46]. Photo-crosslinking of the ellipsoids is then carried out, as in the previous section, significantly increasing their thermal shape stability [18]. To embed the particles in a PDMS matrix, for mechanical stretching, we suspend the PCPMMA spheres in a 25% (w/w) solution of hydroxy terminated PDMS (typical molecular weight $M_{n}=10^{5}$) in hexane. The volume fraction of the PCPMMA spheres in this mixture is low, ϕ≈0.03. Next, a cross-linking agent, trimethylsilyl terminated poly(dimethylsiloxane-co-methylhydrosiloxane) ($M_{n}=950$) and a catalyst [tin(II) 2- ethylhexanoate, ~95%] are added to polymerize the PDMS. The weight fractions of the crosslinking agent and the catalyst are $6\times 10^{-3}$ and $8\times 10^{-3}$, respectively. Immediately after the introduction of the cross-linking agent and the catalyst, the suspension is poured onto a rectangular mold, so that a 1 mm-thick composite rubber film forms. To avoid trapping of hexane bubbles, the curing of this rubber film is carried out under vacuum [14] at 1 mTorr. After a curing time of ≈13 h, the films are post-cured for 2 h in an oven pre-heated to $120{\;}^{\circ }$C. The rubber is then uniaxially stretched to a desired length inside the oven at T = $165{\;}^{\circ }$C, which is above the glass transition temperature of PMMA. To release the ellipsoids, the PDMS matrix is destroyed. For this, it is first swollen in hexane for 24 h. Next, the films are transferred to a mixture of isopropyl alcohol and hexane (5:23 w/w), to which a small amount (0.04%, w/w) of SM is added. This mixture is filled into a hermetically sealed flask, which is placed on a magnetic stirring plate. To help the SM destroy the PDMS matrix, it was found that it was necessary to cut the film into many small fragments. To do this, a piece of ferromagnetic razor was introduced into the flask and agitated by the magnetic field of the stirring plate. When the film is fully degraded, the particles are sedimented by centrifugation and transferred to decalin or hexane. Unfortunately, in addition to degrading the PDMS matrix, SM also destroys parts of the PHSA steric layer. The PHSA molecules are linked to the particle surface by ester bonds, attacked by the SM via a transesterification process [46]. In an attempt to avoid PHSA degradation, we tried destroying the PDMS matrix by sodium tert-butoxide and sodium ethylate. However, these chemicals were unable to degrade the PDMS matrix, even when the process was allowed to proceed for several days.

### 3.5. Fluorescent Staining of the Colloids

The great advantage of our synthesis of PCPMMA colloids is the ability to fluorescently stain the ellipsoids, post-stretching [18], where we employ a swelling/deswelling procedure to introduce rhodamine B chloride into the particles [47]. Significant bleaching of the fluorescent dye occurs during particle stretching, since it is carried at an elevated temperature [14]. However, with the standard PMMA, where the cross-linking is either done during the synthesis of the spheres’, or not done at all, the dye cannot be replenished after particle elongation [3,4,9,46]; the swelling/deswelling procedure would simply make the standard PMMA ellipsoids regain a spherical shape [18]. The PCPMMA particles, photo-crosslinked in their elongated state, do not relax into a spherical shape, even if significantly swollen for loading with the fluorescent dye.

For the swelling/deswelling procedure, we prepared a solution of acetone and cyclohexanone (1:3, by volume), and added to it a small amount (<1%) of rhodamine. Furthermore, 1 mL of this solution was added dropwise, while stirring, to a 2 mL suspension of PCPMMA colloids in dodecane (ϕ≈0.1). The suspension was then mixed for 10 min at ambient temperature. Finally, for the particles to deswell, they were transferred to decalin. Repetitive washing of the particles in decalin was carried out to remove the traces of free fluorescent dye. The same protocol was used for staining of both the spheres and the (photo-crosslinked) ellipsoids.

### 3.6. Formation and Imaging of Fluid Suspensions

We suspend our colloids in a mixture (40:60, by mass) of decalin and TCE. This mixture closely matches the refractive index of our particles. The mass density of this mixture is also very close to that of our colloids, so that the gravitational force acting on the colloids is balanced by the Archimedes force and the particles do not settle on the experimental time scale. For confocal imaging, the sample is loaded into a Vitrocom capillary (VitroCom, Mountain Lakes, NJ, USA) (0.1×2×50 mm or 0.1×1×50 mm) and sealed with an epoxy glue. Our resonant laser scanning confocal setup Nikon A1R (Nikon Instruments Inc., Melville, NY, USA), equipped by an oil-immersed Nikon Plan Apo 100x objective, is capable of obtaining 512 × 512 pixel images at a rate of 15 fps, close to the video rate. For rapid acquisition of 3D stacks of confocal slices through the sample, we mount the objective on a piezo-z stage. With this stage, a collection of 100 slices, separated by 0.3 μm takes only several seconds. At this high data acquisition rate, the diffusion of particles, even for the low density samples, does not matter for structure determination. The lateral digital resolution is set to 0.12 μm/pixel, which is close to the optical resolution of our setup. Our particle tracking codes [3,4,7,48] are based on the PLuTARC implementation [23,49] of the Crocker and Grier algorithm [33] for tracking of the simple spheres. In current work, we find the particle centers within each (quasi-) two-dimensional confocal slice. The results for the spherical PCPMMA are then obtained as an average over many such slices. Structural metrics obtained by this simpler two-dimensional analysis were demonstrated, under similar experimental settings, to almost overlap with data obtained by a full three-dimensional confocal reconstruction [7,35]. The volume fraction of the colloids in the fluids is determined as $\phi ={\left[\sigma \sqrt{\pi /4A_{V}}\right]}^{3}$, where $A_{V}$ is the Voronoi area of a particle, as measured by confocal microscopy, and *σ* is the particle diameter.

## 4. Conclusions

We synthesize photo-crosslinkable PMMA (PCPMMA) colloidal spheres and characterize the size and the size distribution of these particles by SEM and DLS, as well as by the recently-developed [19] ConDDM technique. While an identical particle size is obtained by both DLS and ConDDM, the size obtained by SEM is smaller by ~10%, indicative of particle swelling in organic solvents. We measure the g(r) in dense fluids of PCPMMA spheres and invert these g(r) to obtain the pair potential U(r). Interestingly, the pair potential is demonstrated to incorporate both soft repulsive (probably Coulombic) and attractive (probably, electrostatic-dipolar [22]) contributions. We employ ConDDM measurements to characterize the short-time dynamics in these fluids, for a range of different fluctuation wavelengths. These measurements show the same qualitative behavior as for the hard PMMA spheres. However, the quantitative behavior of the *ϕ*-dependencies is very different, possibly due to the softer and more complex U(r) of our particles. Finally, we demonstrate that the PCPMMA spheres can be stretched and fluorescently-labeled by the swelling/deswelling method, in the stretched state. The stained particles allowed a suspension of bright fluorescent ellipsoids to be formed and imaged by confocal microscopy. Future studies of PCPMMA ellipsoids should allow their sterically-stabilizing PHSA monolayers to be replenished, opening a wide range of new directions for fundamental and application-oriented studies in colloidal science.

## Figures and Tables

**Figure A1:**
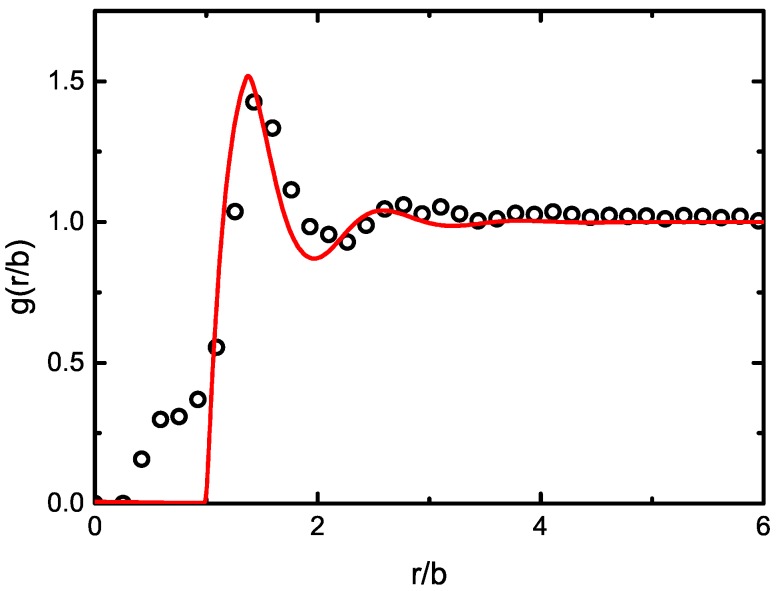
Experimental (open circles), radial distribution function g(r) of a fluid of PCPMMA ellipsoids, at ϕ=0.11. The *r*-values of the experimental data are normalized by the minor axis of the ellipsoids *b*, which have been inflated to account for the non-hard interactions, as described in the text. The **red** curve is the theoretical g(r) of a fluid of hard ellipsoids, based on the Percus–Yevick approximation and fully described elsewhere [4,7].

## Appendix A. PCPMMA Ellipsoids: Preliminary Determination of the Fluid Structure

To reconstruct the structure of a fluid of PCPMMA ellipsoids, we detect the location of each particle in confocal slices through the sample. These locations, for a three-dimensional stack of slices through the sample, are then linked together, as described elsewhere [7]. Thus, the full three-dimensional position of the center of each particle is located; notethat this is a more advanced reconstruction procedure than the two-dimensional one, which is described in the main part of the paper. We use these positions to carry out a Voronoi tesselation of the sample [7]. For the Voronoi volume of a particle being $V_{V}$ and its hard volume being $v_{p}$, the local volume fraction of the particle is $\phi_{L}=v_{p}/V_{V}$. We obtain the distribution of $\phi_{L}$ values across the whole sample. The location of the peak of this distribution corresponds to the volume fraction *ϕ* of the ellipsoids in the fluid.

In order to quantify the local structure of the fluid of prolate PCPMMA ellipsoids at ϕ=0.11, we obtain the radial distribution function g(r); a similar function was used in the main text for the fluids of spheres. The radial distribution is shown in Figure A1 (open circles), where *b* is the short axis of the ellipsoids. Our particles do not interpenetrate; thus, g(r) must be zero for r<b. However, an accurate tracking of the very small ellipsoids employed in the current work is challenging, so that the g(r) exhibits small non-zero values in this range of *r* (see Figure A1). Ellipsoids of a larger size will be employed in the future, completely eliminating these technical issues [7].

We note that the current data are described reasonably well by a theoretical model of hard ellipsoids (described elsewhere [4,7]), provided that all axes of the particles are inflated by ~0.3μm and *ϕ* is rescaled accordingly (red curve in Figure A1). A slightly smaller inflation (0.23 μm) has been previously [4] used for a fluid of common (non-photocrosslinkable) PMMA ellipsoids in a similar solvent. The slightly increased inflation of the current particles, which are also much smaller, indicates that the interparticle potentials in PCPMMA are more complex than with the common PMMA, where the inflation matched the estimated Debye length of the solvent. More detailed studies of the fluids of PCPMMA ellipsoids are currently underway.
